# A prediction nomogram for metabolic syndrome in children: A retrospective study

**DOI:** 10.1371/journal.pone.0334097

**Published:** 2025-10-10

**Authors:** Xiongfeng Pan, Songting Li, Zhiyu Liu, Yan Zhong, Sha Zhao, Jun Qiu

**Affiliations:** 1 Pediatrics Research Institute of Hunan Province, the Affiliated Children’s Hospital of Xiangya School of Medicine, Central South University (Hunan Children’s Hospital), Changsha, China; 2 The School of Pediatrics, Hunan Children’s Hospital, Hengyang Medical School, University of South China, Hengyang, China; 3 Children’s Health Center, The Affiliated Children’s Hospital of Xiangya School of Medicine, Central South University (Hunan children’s hospital), Changsha, China; 4 Changsha Medical University, Changsha, China; 5 The Affiliated Children’s Hospital Of Xiangya School of Medicine, Central South University (Hunan children’s hospital), Hunan Provincial Key Laboratory of Pediatric Orthopedics Changsha, Hunan, China; Rutgers: Rutgers The State University of New Jersey, UNITED STATES OF AMERICA

## Abstract

The aim of this retrospective study was to investigate and develop three screening nomogram models to facilitate early detection and prompt intervention for metabolic syndrome (MS) among children with obesity. We analyzed data from 657 pediatric patients at the Children’s Health Research Institute of Hunan Children’s Hospital, collected between March 1 and September 1, 2020. Participants were stratified into three age groups. Clinically independent predictors were identified using multivariate logistic regression, and then these statistically significant clinical characteristics were recruited to develop a nomogram. The models were designed to estimate individual risk of MS based on clinical characteristics, and subgroup analyses were conducted across age categories. Ten risk factors for MS were identified: rural residence, neck circumference, lymphocyte count, hemoglobin levels, uric acid, C-peptide, alanine aminotransferase, plateletocrit, serum cortisol and fatty liver parameters. In conclusion, rural residence, neck circumference, lymphocyte count, hemoglobin levels, uric acid, serum C-peptide, alanine aminotransferase, platelet count, serum cortisol and fatty liver parameters were identified as independent predictors of MS in children. For each patient, higher total score was associated with increased risk of MS.

## Introduction

Metabolic syndrome (MS) is a complex clinical condition characterized by a cluster of metabolic risk factors, including insulin resistance, obesity, atherogenic dyslipidemia, and hypertension [1]. The rising prevalence of MS has become a major public health challenge worldwide, even among the pediatric population [2]. In recent years, the prevalence of MS in 2020 was estimated at 2.8% for children and 4.8% for adolescents [3]. MS in childhood is associated with an elevated risk of developing type 2 diabetes (T2DM) and cardiovascular diseases (CVD) later in life [4,5]. Consequently, MS has emerged as a critical global pediatric health concern, with its underlying causes still not fully understood.

Although the aetiology and pathogenesis of pediatric MS remain incompletely understood, evidence suggests that clinical factors significantly influence its development. These include gender, age, waist circumference (WC), hyperlipidemia, hyperglycemia, and blood pressure, which are considered critical factors for its development [6–9]. Furthermore, studies reported that place of residence, hemoglobin levels (Hb), glutathione, and total platelet count (PLT) were associated with MS specifically in children [10–13]. Therefore, clinical factors may be important for assessing the risk of pediatric MS.

Pediatric MS exhibits distinct risk factors compared to the adult phenotype, characterized by three unique dimensions: (1) critical developmental windows, exemplified by early-life events such as low birth weight, maternal gestational diabetes, and pubertal timing; (2) behavioral patterns specific to childhood, including excessive screen time, high-frequency sugar-sweetened beverage consumption, and insufficient sleep; and (3) dynamic growth parameters, such as bone age advancement and height velocity-all demonstrating attenuated effects or divergent pathological mechanisms in adult MS [14,15]. This study further identifies novel pediatric-specific biomarkers: neck circumference, lymphocyte count, serum cortisol, and serum C-peptide. Addressing the current research gap in pediatric MS risk stratification models, we pioneer an age-stratified predictive framework integrating these emerging biomarkers with conventional indicators. This methodological innovation offers a novel tool for early MS detection in children, helping to fill an important technological void in pediatric metabolic research.

Nomograms are widely used as statistical visualization tool for screening disease, progression, prognosis, and survival [16–18]. Nevertheless, evidence regarding the association between clinical factors and pediatric MS remains limited, and no risk screening model of clinical factors has been established to screen the occurrence of pediatric MS. Furthermore, metabolic profiles of children across different age groups show inconsistencies.

This study aims to utilise clinical factors to explore the mechanisms of pediatric MS and develop screening models using nomograms. These models will include clinical factors for children aged 6–9 years, 10–18 years, and a combined model for both age groups, facilitating early screening and timely management of pediatric MS in obese children. These models may also lay the foundation for future research on the pathogenesis of pediatric MS, and provides clinicians with a simple and intuitive tool for pediatric MS practical prediction.

## Materials and methods

### Study participants

This retrospective study used data from pediatric patients attending Institute of Child Health, Hunan Children’s Hospital (Changsha, China). Six hundred and fifty-seven children were recruited from March 1st to September 1st in 2020. Inclusion criteria for the participants were as follows: (1) 6 ≤ age ≤ 18; (2) their parents signed a written informed consent form. Exclusion criteria were as follows: (1) congenital inherited metabolic diseases; (2) neurological disorders; (3) endocrine diseases; (4) infectious diseases. MS was diagnosed in accordance with the Pediatrics Branch of the Chinese Medical Society Guidelines [14], which were established by the Pediatrics Branch of the Chinese Medical Association in The Definition of MS and Prophylaxis and Treatment Proposal in Chinese Children and Adolescents.

### Data collection

The following clinical characteristics were collected for all children: residence, gender, age, weight, pulse, height (Ht), body mass index (BMI), hip circumference (HC), neck circumference, etc. Laboratory and metabolic parameters were obtained from electronic medical records: Hb, PLT, cholesterol, serum cortisol, and serum C-peptide, alanine aminotransferase (ALT), aspartate aminotransferase (AST), uric acid (UA), insulin (INS), total protein (TP), globulin (GLOB), creatinine (Cr), fatty liver parameters, procalcitonin (PCT), liver stiffness (LS), hematocrit (HCT), white blood cell count (WBC), red blood cell count (RBC), etc.

A comprehensive data cleaning protocol was applied to all variables. This process involved: (1) Validity cleaning of variables: encompassing the identification and correction of logical errors, and the detection of outliers (determined through literature review and expert consultation), with observations containing outliers subsequently corrected or removed; (2) Integrity cleaning of variables: primarily addressing the handling of missing values. For specific variables such as missing age or gender, information from ID numbers can be utilized for imputation. Missing age can be derived by calculating the subject’s birth date from the ID number and subtracting it from the survey date. Missing gender can be determined using the second-to-last digit of the ID number, where an odd digit represents male and an even digit represents female. Simultaneously, for variables with minimal missingness (e.g., less than 5%), mean imputation was applied. The study included a sensitivity analysis.

For information bias control, we employed structured data validation protocols: (1) Dual independent data entry with cross-verification using EpiData software (version 3.1), (2) Random audits of 15% medical records showing 98% inter-rater agreement (κ = 0.92), and (3) Logic checks for outlier values (e.g., biologically implausible anthropometric measurements).

### Ethical statement

Hunan Children’s Hospital Ethics Committee approved the study, and participants provided oral and written informed consent prior to the use of their serum samples in the analyses. The study protocol was approved by the Hunan children’s Hospital Ethics Research Committee (XYGW201804).

### Construction and evaluation of the prediction model

In our study, univariate and multivariate logistic regression analyses were performed to identify factors associated with MS in children. Variables directly related to the definition of MS were excluded, while all other variables were included in the univariate analysis. All variables meeting the prespecified significance threshold (p < 0.05) in univariate analysis were included in the preliminary multivariable regression model. We subsequently employed a forward stepwise selection strategy, informed by clinical expertise, to construct the final multivariable predictive model. Predictors retained in this final model were utilized for nomogram construction. Systematic assessment of multicollinearity among predictors was performed both before and after developing the final multivariable model to ensure stability and interpretability of the coefficients. Variance inflation factors (VIF) and tolerance statistics were calculated, with VIF > 10 indicating substantial multicollinearity requiring remediation. Where severe multicollinearity was detected (e.g., VIF > 10), affected variables were excluded. All predictors in the final model demonstrated VIF < 10, Complete diagnostic metrics, including variable-specific VIF values, have been provided in the Supplementary Material.

Subsequently, we applied Least Absolute Shrinkage and Selection Operator (LASSO) regression analysis, using the same set of variables as in the logistic regression model. All continuous variables were standardized prior to being entered into the model. The regularization parameter λ was determined through 10-fold stratified cross-validation, with the selection criterion being minimization of the cross-validated mean squared error (λ.min). To achieve optimal predictive performance, we constructed the final predictive model using λ.min.

Independent clinical characteristics were evaluated through multivariate logistic regression, and then these clinical characteristics were recruited to develop a nomogram. A nomogram model was designed to asses MS, and further conduct subgroup analysis on different ages. The receiver operating characteristic (ROC) curve, the area under the ROC curve (AUC), the concordance index (C-index), and the calibration curve were used to evaluate the predictive accuracy and consistency of the nomogram model. Decision curve analysis (DCA) reflects the net benefit of the model to patients. Both discrimination and calibration were evaluated through 1000 resamples.

### Statistical analysis

For demographic and clinical parameters with continuous variables, normality test was conducted through the Kolmogorov-Smirnov test (K-S test). The normal distribution variables were described using the mean±standard deviation (X ± SD), and the t-test was used for the MS group and the control group comparison. For the skewed distribution variables, it was described using the median (M) and interquartile range (P25, P75), and the Mann-Whitney U test was used for intergroup comparison. Count data were expressed as a ratio, the χ^2^ test or Fisher’s exact probability method was used for intergroup comparison. A two-tailed P value <0.05 was considered statistically significant. All statistical analyses were performed using R version 4.3.2 and rms, glmnet, pROC, ggplot2, and dca packages.

## Results

### Study selection

**Fig 1:**
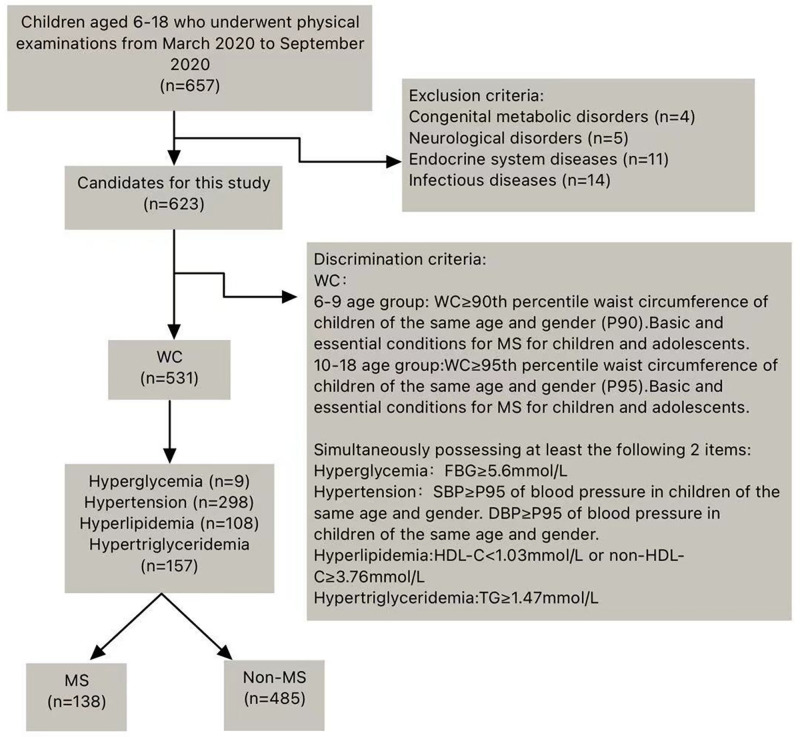
Study design overview. Study populations were recruited at the first hospital admission, at which time relevant data were collected. Flowchart of the populations included in our final analysis. WC = Waist Circumference; GLU = Glucose; TG = Triglyceride; MS = Metabolic Syndrome; SBP = Systolic Blood Pressure; DBP = Diastolic Blood Pressure; FBG = Fasting Blood Glucose.

The retrospective study initially included 657 children from the Hunan Children’s Hospital. Thirty-four children did not meet the inclusion criteria, 4 children had congenital inherited metabolic diseases, 5 had neurological disorders, 11 had endocrine diseases, and 14 had infectious diseases. Finally, 623 children were enrolled in this study (Fig 1).

### Characteristics of the study population

Among the 623 children, 138 (22.15%) children were classified as the MS group and 485 as the non-MS group. The detection rate of MS among boys and girls were 22.37% and 21.56%. The detection rate of MS in the 6–9 age group is 15.38%, and in the 10–18 age group it is 25.87% (S1 Table).

Compared to the non-MS group, the MS group had higher values for Ht (p < 0.001), Wt (p < 0.001), WC (p < 0.001), neck circumference (p < 0.001), ALT (p < 0.001), and AST (p = 0.001) (Table 1), MS group children aged 6–9 years had higher values of Ht (p = 0.020), Wt (p = 0.002), HC (p = 0.005), number of basophils (p = 0.016), UA (p = 0.012), and INS (p = 0.010) than the non-MS group (S2 Table). For the 10–18 age group, the MS group had higher values of TP (p < 0.001), GLOB (p = 0.001), PLT (p = 0.001), and Cr (p = 0.002) than the non-MS group (S3 Table).

**Table 1:** Statistical description of the original dataset in 6-18 age group.

Variables	Non-MS (n = 485) M(P25,P75)/N(%)	MS (n = 138) M(P25,P75)/N(%)	Total (n = 623) M(P25,P75)/N(%)	*t/c* ^*2*^ */Z*	*P-value*
NAFLD				20.85	<0.001
+	112(23.1)	59(42.8)	171(27.4)		
–	373(76.9)	79(57.2)	452(72.6)		
Age	10.00(9.00,12.00)	11.00(9.75,13.00)	10.00(9.00,12.00)	−3.97	<0.001
Residence				6.76	0.009
Urban	382(78.8)	94(68.1)	476(76.4)		
Rural	103(21.2)	44(31.9)	147(23.6)		
Ht	142.60 ± 12.24	148.07 ± 11.39	143.82 ± 12.26	4.70	<0.001
Wt	48.40(39.95,58.70)	57.05(47.98,67.23)	50.60(41.30,61.10)	−6.36	<0.001
SBP	113.00(104.50,122.50)	125.00(116.75,131.00)	116.00(107.00,126.00)	−7.74	<0.001
SBP+				66.36	<0.001
+	157(32.4)	98(71)	255(40.9)		
–	328(67.6)	40(29)	368(59.1)		
DBP	65.00(60.00, 71.50)	74.00(65.75, 79.00)	66.00(61.00,74.00)	−7.28	<0.001
DBP+				32.61	<0.001
+	80(16.5)	54(39.1)	134(21.5)		
–	405(83.5)	84(60.9)	489(78.5)		
Hypertension				89.53	<0.001
+	183(37.7)	115(83.3)	298(47.8)		
–	302(62.3)	23(16.7)	325(52.2)		
WC	81(72.25,87.35)	86.55(81.58,93.63)	82.10(74.10,89.40)	−7.01	<0.001
WC+				30.71	<0.001
+	393(81.0)	138(100)	531(85.2)		
–	92(19)	0(0.0)	92(14.8)		
HC	87.36 ± 10.67	93.67 ± 9.72	88.76 ± 10.78	6.25	<0.001
Neck circumference	31.80(29.50,33.80)	34.00(32.00,36.13)	32.10(30.00,34.50)	−7.25	<0.001
WBC	7.12(6.05, 8.12)	7.39(6.42, 8.95)	7.26(6.10,8.25)	−2.60	0.009
NEU	3.80(3.00,4.63)	3.97(3.23,4.99)	3.84(3.04,4.71)	−2.06	0.040
Lymphocyte count	2.65(2.21,2.97)	2.72(2.35,3.21)	2.68(2.24,3.03)	−2.50	0.012
RBC	4.89(4.64,5.08)	4.98(4,72,5.25)	4.90(4.65,5.12)	−3.28	0.001
Hb	134.00(128.00,138.00)	137.00(131.00,144.00)	134.00(129.00,140.00)	−5.13	<0.001
HCT	39.93(38.20,41.40)	40.75(39.35,42.95)	39.93(38.40,41.70)	−4.85	<0.001
PCT	0.32(0.28, 0.35)	0.34(0.30, 0.38)	0.32(0.28,0.35)	−3.51	<0.001
PLT	312.00(272.00, 343.50)	327.50(282.50, 376.25)	313.18(275.00,353.00)	−3.04	0.002
BAKP	224.69(220.00, 230.00)	221.83(215.00, 230.00)	224.69(217.14,230.00)	−2.06	0.039
TP	70.80(68.20,73.40)	72.00(69.98,74.00)	71.03(68.50,73.50)	−4.12	<0.001
GLOB	28.10(26.05,29.70)	28.95(27.40,30.90)	28.11(26.40,30.00)	−4.27	<0.001
A/G	1.55(1.40, 1.67)	1.49(1.37, 1.61)	1.53(1.40,1.65)	−2.71	0.007
ALT	20.20(14.90, 32.49)	31.80(19.05, 54.63)	22.00(15.70,35.20)	−5.75	<0.001
AST	22.80(19.30, 26.70)	24.60(20.15, 32.83)	23.30(19.40,27.70)	−3.21	0.001
AST/ALT	1.07(0.80, 1.36)	0.78(0.56, 1.08)	1.00(0.72,1.30)	−6.24	<0.001
GLU+				8.04	0.005
+	3(0.6)	6(4.3)	9(1.4)		
–	482(99.4)	132(95.7)	614(98.6)		
BUN	4.17(3.58,4.79)	4.01(3.29,4.62)	4.17(3.53,4.76)	−2.07	0.039
Cr	43.00(38.15,47.00)	45.80(39.93,51.97)	43.60(38.40,48.40)	−3.84	<0.001
UA	352.00(306.00,403.00)	387.00(341.75,464.00)	362.00(313.00,413.00)	−5.66	<0.001
TG	0.95(0.73,1.26)	1.83(1.52,2.36)	1.06(0.77,1.47)	−13.34	<0.001
TG+				302.15	<0.001
+	44(9.1)	113(81.9)	157(25.2)		
–	441(90.9)	25(18.1)	466(74.8)		
CHOL	3.79(3.39,4.17)	4.03(3.64,4.61)	3.86(3.43,4.25)	−4.53	<0.001
HDL-C	1.28(1.15,1.44)	1.06(0.95,1.24)	1.26(1.10,1.40)	−8.96	<0.001
LDL-C	2.05(1.75,2.37)	2.27(1.91,2.78)	2.10(1.78,2.46)	−4.02	<0.001
Lipid				143.96	<0.001
+	37(7.6)	71(51.4)	108(17.3)		
–	448(92.4)	67(48.6)	515(82.7)		
Hepatic hemoglobin	15.33(14.82,15.88)	15.61(15.17,16.20)	15.45(14.90,15.90)	−3.97	<0.001
HbA1c	0.75(0.71,0.78)	0.76(0.72,0.79)	0.75(0.71,0.78)	−3.11	0.002
Serum cortisol	9.06(6.99,11.08)	9.80(7.26,12.48)	9.29(7.03,11.35)	−2.47	0.014
INS	17.64(13.00,23.65)	24.80(16.91)	19.05(13.52,26.16)	−6.28	<0.001
C-Peptide	2.68(2.14,3.19)	3.36(2.66,4.08)	2.80(2.24,3.37)	−7.43	<0.001
LS	5.10(4.40,5.60)	5.15(4.83,5.90)	5.15(4.50,5.70)	−3.16	0.002
Fatty liver parameters	230.90(220.00,238.03)	236.82(229.48,251.68)	231.20(221.50,240.20)	−5.66	<0.001

NAFLD = Non-alcoholic fatty liver disease; Ht = Height; Wt = Weight; SBP = Systolic blood pressure; DBP = Diastolic blood pressure; WC = Waist circumference; HC = Hip circumference; WBC = White blood cell count; NEU = Number of neutrophils; LYM = Lymphocyte count; RBC = Red blood cell; Hb = Hemoglobin; HCT = Hematocrit; PCT = Plateletocrit; PLT = Total platelet count; BAKP = Bone alkaline phosphatase; TP = Total protein; GLOB = Globulin; A/G = Albumin/Globulin; ALT = Alanine aminotransferase; AST = Aspartate aminotransferase; AST/ALT = Aspartate aminotransferase/Alanine aminotransferase; GLU = Glucose; BUN = Blood Urea Nitrogen; Cr = Creatinine; UA = Uric acid; TG = Triglyceride; CHOL = Cholesterol; HDL-C = High-density lipoprotein cholesterol; LDL-C = Low-density lipoprotein cholesterol; HbA1c = Glycated hemoglobin; INS = Insulin; + = yes; - = no; LS = Liver Stiffness.

### Variable selection affecting MS

**Fig 2:**
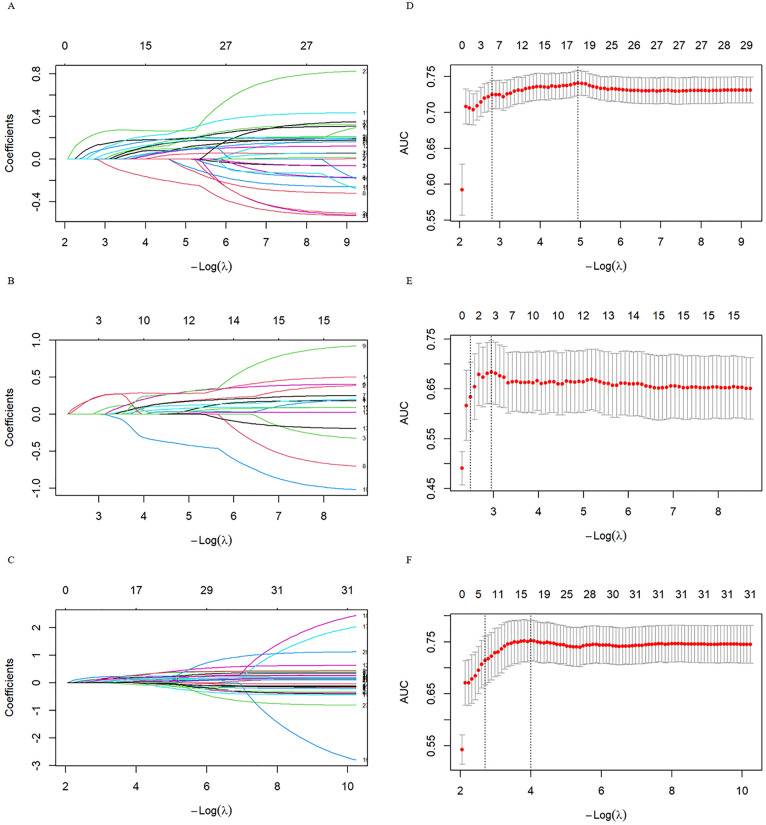
LASSO coefficient paths and optimal λ selection. Panels A, B, and C: Each line represents a different predictor variable in the LASSO regression model; Panels D, E, and F: The lambda.min is highlighted, indicating the optimal level of penalization for the LASSO model. Panels A and D: Correspond to the 6-18 age group; Panels B and E: Correspond to the 6-9 age group; Panels C and F: Correspond to the 10-18 age group.

Sixty-four variables were subjected to univariate regression analysis, yielding 29 statistically significant variables (P < 0.05) for the 6−18 age group (S4 Table). To mitigate multicollinearity, variables with a VIF > 10 were excluded (S5 Table). Six independent predictors were included in the nomogram model: residence (P = 0.023, OR 1.729, 95% CI 1.078–2.773), neck circumference (P = 0.014, OR 1.101, 95% CI 1.020–1.188), lymphocyte count (P = 0.042, OR 1.342, 95% CI 1.010–1.783), Hb (P = 0.002, OR 1.041, 95% CI 1.015–1.068), UA (P = 0.041, OR 1.003, 95% CI 1.000–1.006) and serum C-peptide (P = 0.004, OR 1.372, 95% CI 1.106–1.701) (Table 2). Through this rigorous analytical process, we successfully refined the collected factors to 17 statistically significant key indicators (Fig 2). These critical predictors included: residence, neck circumference, lymphocyte count, Hb, UA, serum C-peptide, fatty liver parameters, AST/ALT ratio, serum cortisol, PCT, TP, GLOB, LS, HCT, WBC, RBC, and PLT (Table 3). All retained variables demonstrated non-zero coefficients in the final LASSO model.

**Table 2:** Binary logistic regression analysis of MS.

Variables	β	S.E.	Waldχ²	OR(95%Cl)	P-value
**6-18 age group**					
Residence	.547	.241	5.156	1.729(1.078 ~ 2.773)	.023
Neck circumference	.096	.039	6.078	1.101(1.02 ~ 1.188)	.014
Lymphocyte count	.294	.145	4.123	1.342(1.01 ~ 1.783)	.042
Hb	.040	.013	9.406	1.041(1.015 ~ 1.068)	.002
UA	.003	.001	4.184	1.003(1 ~ 1.006)	.041
C-Peptide	.316	.110	8.284	1.372(1.106 ~ 1.701)	.004
**6-9 age group**					
ALT	.024	.009	6.877	1.024(1.006 ~ 1.043)	.009
C-Peptide	.786	.274	8.230	2.194(1.283 ~ 3.752)	.004
**10-18 age group**					
Residence	1.093	.289	14.326	2.984(1.694 ~ 5.257)	.000
Neck circumference	.151	.045	11.397	1.163(1.065 ~ 1.27)	.001
HB	.057	.016	12.822	1.059(1.026 ~ 1.093)	.000
PLT	.006	.002	8.395	1.006(1.002 ~ 1.009)	.004
Serum cortisol	.091	.031	8.483	1.095(1.03 ~ 1.164)	.004
Fatty liver parameters	.019	.008	6.348	1.019(1.004 ~ 1.034)	.012

OR = odds ratio; CI = confidence interval.

**Table 3:** Coefficients and lambda.min value of the LASSO regression.

Variable	Variable Coefficient	Lambda min
6-18 age group		0.007121433
Residence	0.18800954	
Neck circumference	0.19169185	
Lymphocyte count	0.1773037	
Hb	0.27575272	
UA	0.19767621	
C.Peptide	0.2654306	
Fatty liver parameters	0.17079205	
AST/ALT	-0.22838751	
Serum cortisol	0.16600645	
PCT	0.10675797	
TP	0.10379101	
GLOB	0.09131056	
LS	0.08711455	
HCT	0.08439535	
WBC	-0.07510753	
RBC	-0.05764381	
PLT	0.04639586	
6-9 age group		0.05239078
ALT	0.22644475	
C-Peptide	0.23450968	
Wt	0.02733853	
10-18 age group		0.01830692
Residence	0.295074738	
Neck circumference	0.190155964	
HB	0.268961961	
PLT	0.143469092	
Serum cortisol	0.28234827	
Fatty liver parameters	0.178507867	
C-Peptide	0.236628741	
UA	0.160590326	
PCT	0.044466139	
TP	0.117767285	
LS	0.080275549	
AST/ALT	-0.067650926	
HCT	0.054173811	
Lymphocyte count	0.031794523	
GLOB	0.030189339	
Hepatic hemoglobin	0.007051683	
FLD	-0.006563369	

### Subgroup of variable selection affecting MS in age-stratified children

Specifically, for the 6–9 age group, 15 variables (P < 0.05) were identified, as shown in S6 Table. Using LASSO regression analysis, we refined the initial set of factors to three key predictors (Table 3). Variables exhibiting VIF > 10 were excluded to mitigate multicollinearity (S7 Table). Among the three significant variables from the 6–9 age group: ALT (P = 0.009, OR 1.024, 95%CI 1.006–1.043) and serum C-peptide (P = 0.004, OR 2.194, 95%CI 1.283–3.752), which were included in the nomogram model (Table 3).

In the 10−18 age group, 31 significant variables were identified (P < 0.05), as presented in S8 Table. The LASSO regression analysis refined the initial set of factors to seventeen key predictors (Table 3). Variables exhibiting VIF > 10 were excluded to mitigate multicollinearity (S9 Table). Similarly, for the 10−18 age group, six factors associated with MS were identified: residence (P < 0.001, OR 2.984, 95%CI 1.694–5.257), neck circumference (P = 0.001, OR 1.163, 95%CI 1.065–1.270), Hb (P < 0.001, OR 1.059, 95%CI 1.026–1.093), PLT (P = 0.004, OR 1.006, 95%CI 1.002–1.009), serum cortisol (P = 0.004, OR 1.095, 95%CI 1.030–1.164), and fatty liver parameters (P = 0.012, OR 1.019, 95%CI 1.004–1.034), all of which were incorporated into the predictive model (Table 2).

### Risk prediction nomogram development

**Fig 3:**
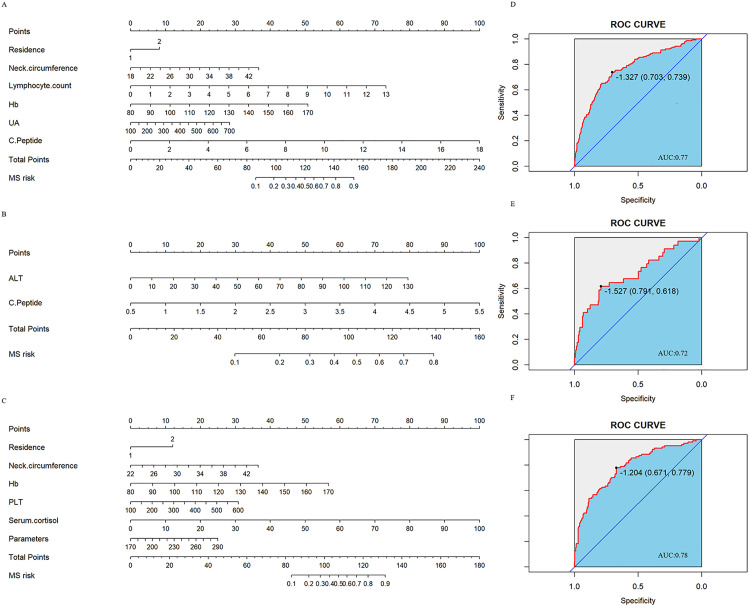
Nomogram and ROC curves for the prediction of MS in children. Panels A-C display the nomograms, while Panels D-F present the ROC curves. Visualization of the nomogram model. Variables are displayed on the left side, whereas scores are shown on the right side. The total score is calculated by adding the values of each variable. ROC of the nomogram model. The x-axis represents 1-specificity, whereas the y-axis represents sensitivity. ROC = receiver operating characteristic; AUC = area under the ROC curve. Residence: 1 = Urban; 2 = Rural; Parameters = fatty liver parameters. Panels A and D: Correspond to the 6–18 age group; Panels B and E: Correspond to the 6–9 age group; Panels C and F: Correspond to the 10–18 age group.

The nomogram model was constructed based on the above 6 factors for the 6–18 age group (Fig 3A). The total score, obtained by summing the individual scores of the residence, neck circumference, lymphocyte count, Hb, UA and C-Peptide. A higher total score on the nomogram indicated a greater risk of MS for the 6–18 age group.

A nomogram model for the 6–9 age group was constructed based on the above 2 factors (Fig 3B). The total score obtained by adding individual scores of the ALT and C-Peptide, a higher total score indicated a greater probability of MS in children aged 6–9. The nomogram model for the 10–18 age group was constructed based on the six factors (Fig 3C). The total score obtained by adding the individual scores for residence, neck circumference, Hb, PLT, serum cortisol and fatty liver parameters in children aged 10–18.

The overall predictive model demonstrated good discrimination, with an AUC of 0.77, a sensitivity of 73.90%, and a specificity of 70.30% (Fig 3D). For the 6–9 age group, the AUC was 0.72, a sensitivity of 61.80%, and a specificity of 79.10%, indicating good discrimination (Fig 3E). For the 10–18 age group, the AUC was 0.78, a sensitivity of 77.90%, and a specificity of 67.10%, also suggesting good discrimination (Fig 3F).

**Fig 4:**
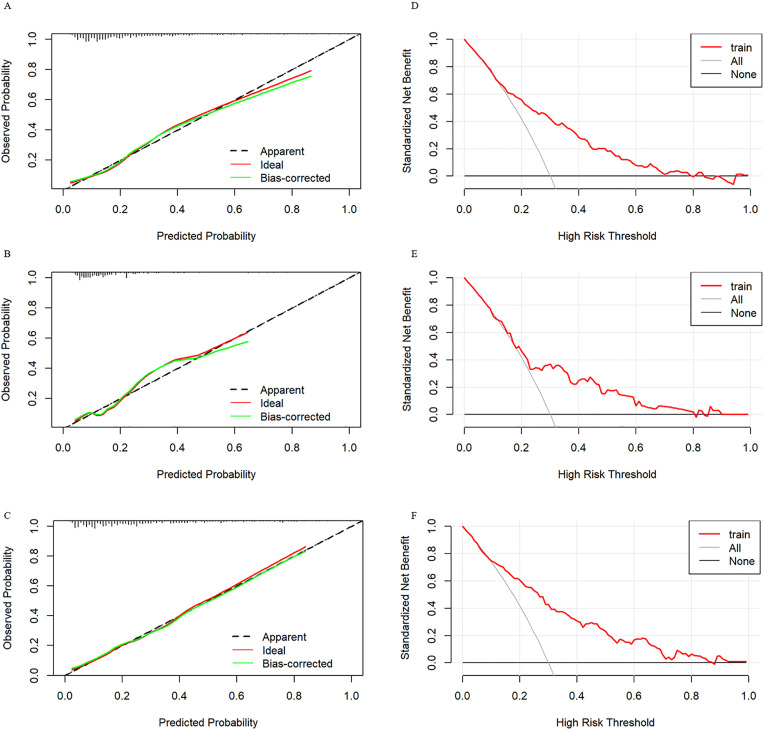
Calibration curve and DCA for predicting the probability of MS in children. Panels A, B, and C present calibration curves, while Panels D, E, and F DCA plots. The x-axis of the calibration curves represents the probability of the ideal level, and the y-axis represents the actual level of the nomogram model. The black dashed line represents perfect prediction based on actual probability. The ideal diagonal line represents the prediction of the nomogram model. The closer the interval is to the black dashed line, the better the performance of the nomogram model. The x-axis of the DCA represents the threshold probability, and the y-axis represents the net benefit of the nomogram model. The black line represents the net benefit when all participants fail to achieve the ideal level. In contrast, the grey line represents the net benefit when all participants achieve the ideal level. The red line represents the net benefit according to the model’s predictions. DCA = Decision curve analysis. Panels A and D: Correspond to the 6-18 age group; Panels B and E: Correspond to the 6-9 age group; Panels C and F: Correspond to the 10-18 age group.

Furthermore, calibration curves demonstrated close agreement between predicted and observed probabilities, affirming the model’s excellent calibration (Figs 4A-C). Additionally, DCA provided evidence of substantial clinical benefit associated with the predictive model (Figs 4D-F).

In the 6–18 age group, key predictors including neck circumference (p < 0.015), Hb (p < 0.007), and UA (p < 0.001) remained robust across all sensitivity analyses (S1 Fig). Within the 6–9 age group, key predictors such as ALT (p < 0.006) were consistently robust in all sensitivity analyses (S2 Fig). For the 10–18 age group, key predictors including Hb (p < 0.015), PLT (p < 0.001), serum cortisol (p < 0.015), and fatty liver parameters (p < 0.004) demonstrated robustness throughout all sensitivity analyses, supporting the reliability of the primary findings (S3 Fig).

## Discussion

This study identified ten risk factors associated with MS in children: rural living, neck circumference, lymphocyte count, Hb levels, UA, serum C-peptide, ALT, PLT, serum cortisol and fatty liver parameters. Among these, rural residence, hemoglobin, and PLT have been linked to MS, while there is limited research on the relationship between neck circumference, lymphocyte count, serum cortisol, and serum C-peptide in children with obesity. Additionally, no prior studies have developed a risk screening model for MS in this population. Notably, serum C-peptide was identified as a significant predictor in both the 6–18 and 6–9 age groups. Three factors rural residence, neck circumference and hemoglobin were identified as a significant predictor in both the 6–18 and 10–18 age groups. The lymphocyte count and UA were identified as a significant predictor in the 6–18 age group, ALT was identified as a significant predictor in the 6–9 age group, and PLT, serum cortisol and fatty liver parameters were identified as a significant predictor in the 10–18 age group.

Research indicates that factors such as place of residence and Hb levels are associated with MS in individuals aged 6–18. Specifically, living in a rural area is identified as a risk factor, aligning with the findings of Lee et al. [13], Rus et al. [19], and Yu et al. [20], but differing from the research conducted by Yang et al. [21] and Xu et al. [22]. This discrepancy may arise from regional differences in economic conditions, educational approaches, lifestyle habits, and cultural influences between urban and rural settings. Additionally, rural areas have been associated with specific dietary patterns, including higher carbohydrate and sodium intake [13]. Blood biomarkers such as Hb, serum cortisol, and C-peptide have been linked to CVD. With the increasing prevalence MS, the risk of stroke, CVD, and mortality also rise, suggesting that the association between blood biomarkers and MS may reflect early stages of CVD development [19]. Although CVD is rare in childhood, the atherosclerotic process can begin early and is closely tied to lipid levels.

The 6–18 age group can be divided into two subgroups for analysis. In the 6–9 age group, research indicates a strong correlation between elevated ALT levels and MS, with children affected by MS showing the higher ALT levels compared to those without MS. Impaired fasting glucose was only linked to increased ALT levels, which aligns with our findings [10]. In the 10–18 age group, PLT was associated with MS, and a study has shown that biomarkers like RBC, Hb, PLT, and RBC distribution width are elevated in MS patients [11,23]. Consistent with Yang et al., inflammatory markers such as leukocytes and platelets, specifically total leukocytes, lymphocytes, monocytes, neutrophils, PLT, platelet pressure, and platelet distribution width, are statistically linked to the presence of MS [24]. Platelets are crucial in the development of atherothrombosis. In vivo platelet activation has been observed in individuals with CVD, including T2DM, insulin resistance, hypertension, hyperlipidemia, and smoking, indicating that this activation may contribute to CVD [25]. Conversely, using antiplatelet therapy to inhibit platelets is an important approach to mitigate CVD. In T2DM, several abnormalities in platelet characteristics have been identified, which help define ‘enhanced platelet activation,’ including changes in membrane receptors and increased release of thromboxane metabolites in vivo.

Serum C peptide represents as a risk factor across various age groups. It is a protein hormone produced by the β-cells of the pancreatic islets, playing a crucial role in glucose homeostasis by facilitating glycogen breakdown, and gluconeogenesis, and enhancing glucose uptake and utilization in peripheral tissues, thereby helping to lower blood glucose. It is also a key indicator for assessing insulin secretion function. Low levels of serum C-peptide are associated with a higher risk of coronary heart disease and cerebral infarction, as well as increased blood lipid levels. Low serum C-peptide adversely influences the progression of T2DM [26]. Research indicates that elevated fasting serum C-peptide levels are a significant marker for cardiovascular risk linked to MS [27].

Furthermore, calibration curve analysis revealed that the correlation between predicted and actual probabilities of MS was stronger in the three age groups studied, with the 10–18 age group showing a closer alignment to the diagonal, indicating superior predictive accuracy of the model in this pediatric population.

However, some limitations of this study should be acknowledged. First, to minimize multicollinearity among variables, this study employed a one-factor combined with multifactorial analysis, which is suitable for studies with fewer variables. Although the VIF test was introduced to the methodology. For studies with more variables, advanced analytical techniques like LASSO analysis should be considered. As this is a retrospective study, establishing causal relationships from biomarker findings is challenging. Additionally, the study focused solely on children in China, which may limit its applicability to other ethnic groups. This retrospective cohort study acknowledges inherent limitations including potential selection bias and information bias. To mitigate selection bias, consecutive enrollment of all eligible children presenting at our institution between March 2020 and September 2020 was implemented, achieving an exclusion rate <5% (34/657). Moreover, while sensitivity analysis can assess the stability of the results, its findings are not absolute and should be interpreted with caution. Consequently, even when sensitivity analyses indicated instability for some variables included in the model, our modeling decisions were ultimately driven by clinical relevance and biological plausibility, these analyses may introduce information bias.

## Conclusions

In conclusion, living in rural areas, neck circumference, lymphocyte count, Hb, UA, serum C-peptide, ALT, PLT, serum cortisol and fatty liver parameters were identified as independent predictors of MS in children. For each child, higher total points reflected a greater risk of MS. The visual and personalized model of clinical factors provides clinicians with a simple and intuitive tool for the early detection and identification of children with MS.

## Supporting information

S1 Fig Sensitivity analyses for the 6–18 age group. (TIF)

S2 Fig Sensitivity analyses for the 6–9 age group. (TIF)

S3 Fig Sensitivity analyses for the 10–18 age group. Parameters = fatty liver parameters.
(TIF)

S1 Table Statistical description of the original dataset in 6–18 age group. GR% = Granulocyte ratio; LY% = Lymphocyte ratio; EOS% = Eosinophil ratio; MCV = Mean red cell volume; MCHC = Mean corpuscular hemoglobin concentration; MPV = Mean platelet volume; RDW-SD = Red blood cell volume distribution width; PDW = Platelet distribution width; T-bill = Total bilirubin; D-BIL = Direct bilirubin; IBIL = Indirect bilirubin; ALB = Albumin; TBA = Total biliary acid; GLU = Glucose; HbA1c% = Glycosylated hemoglobin ratio; H = Hardness; FLD = Fatty liver disease; POS = Probability Of Success.
(XLSX)

S2 Table Statistical description of the original dataset in 6–9 age group. GR% = Granulocyte ratio; LY% = Lymphocyte ratio; EOS% = Eosinophil ratio; MCV = Mean red cell volume; MCHC = Mean corpuscular hemoglobin concentration; MPV = Mean platelet volume; RDW-SD = Red blood cell volume distribution width; PDW = Platelet distribution width; T-bill = Total bilirubin; D-BIL = Direct bilirubin; IBIL = Indirect bilirubin; ALB = Albumin; TBA = Total biliary acid; GLU = Glucose; HbA1c% = Glycosylated hemoglobin ratio; H = Hardness; FLD = Fatty liver disease; POS = Probability Of Success; LS = Liver Stiffness.
(XLSX)

S3 Table Statistical description of the original dataset in 10–18 age group. GR% = Granulocyte ratio; LY% = Lymphocyte ratio; EOS% = Eosinophil ratio; MCV = Mean red cell volume; MCHC = Mean corpuscular hemoglobin concentration; MPV = Mean platelet volume; RDW-SD = Red blood cell volume distribution width; PDW = Platelet distribution width; T-bill = Total bilirubin; D-BIL = Direct bilirubin; IBIL = Indirect bilirubin; ALB = Albumin; TBA = Total biliary acid; GLU = Glucose; HbA1c% = Glycosylated hemoglobin ratio; H = Hardness; FLD = Fatty liver disease; POS = Probability Of Success; LS = Liver Stiffness.
(XLSX)

S4 Table Results of the univariate logistic regression analysis of risk factors for 6–18 age group. GR% = Granulocyte ratio; LY% = Lymphocyte ratio; EOS% = Eosinophil ratio; MCV = Mean red cell volume; MCHC = Mean corpuscular hemoglobin concentration; MPV = Mean platelet volume; RDW-SD = Red blood cell volume distribution width; PDW = Platelet distribution width; T-bill = Total bilirubin; D-BIL = Direct bilirubin; IBIL = Indirect bilirubin; ALB = Albumin; TBA = Total biliary acid; GLU = Glucose; HbA1c% = Glycosylated hemoglobin ratio; H = Hardness; FLD = Fatty liver disease; POS = Probability Of Success; LS = Liver Stiffness.
(XLSX)

S5 Table VIF results for the 6–18 age group. VIF = Variance Inflation Factor.
(XLSX)

S6 Table Results of the univariate logistic regression analysis of risk factors for 6–9age group. GR% = Granulocyte ratio; LY% = Lymphocyte ratio; EOS% = Eosinophil ratio; MCV = Mean red cell volume; MCHC = Mean corpuscular hemoglobin concentration; MPV = Mean platelet volume; RDW-SD = Red blood cell volume distribution width; PDW = Platelet distribution width; T-bill = Total bilirubin; D-BIL = Direct bilirubin; IBIL = Indirect bilirubin; ALB = Albumin; TBA = Total biliary acid; GLU = Glucose; HbA1c% = Glycosylated hemoglobin ratio; H = Hardness; FLD = Fatty liver disease; POS = Probability Of Success; LS = Liver Stiffness.
(XLSX)

S7 Table VIF results for the 6–9 age group. VIF = Variance Inflation Factor.
(XLSX)

S8 Table Results of the univariate logistic regression analysis of risk factors for 10–18 age group. GR% = Granulocyte ratio; LY% = Lymphocyte ratio; EOS% = Eosinophil ratio; MCV = Mean red cell volume; MCHC = Mean corpuscular hemoglobin concentration; MPV = Mean platelet volume; RDW-SD = Red blood cell volume distribution width; PDW = Platelet distribution width; T-bill = Total bilirubin; D-BIL = Direct bilirubin; IBIL = Indirect bilirubin; ALB = Albumin; TBA = Total biliary acid; GLU = Glucose; HbA1c% = Glycosylated hemoglobin ratio; H = Hardness; FLD = Fatty liver disease; POS = Probability Of Success; LS = Liver Stiffness.
(XLSX)

S9 Table VIF Results for the 10–18 Age Group. VIF = Variance Inflation Factor.
(XLSX)
