# Supplementary figures and images for: A prediction nomogram for metabolic syndrome in children: A retrospective study
Source: PLoS One. 2025 Oct 10;20(10):e0334097. doi: 10.1371/journal.pone.0334097 (PMC12513670; doi:10.1371/journal.pone.0334097)

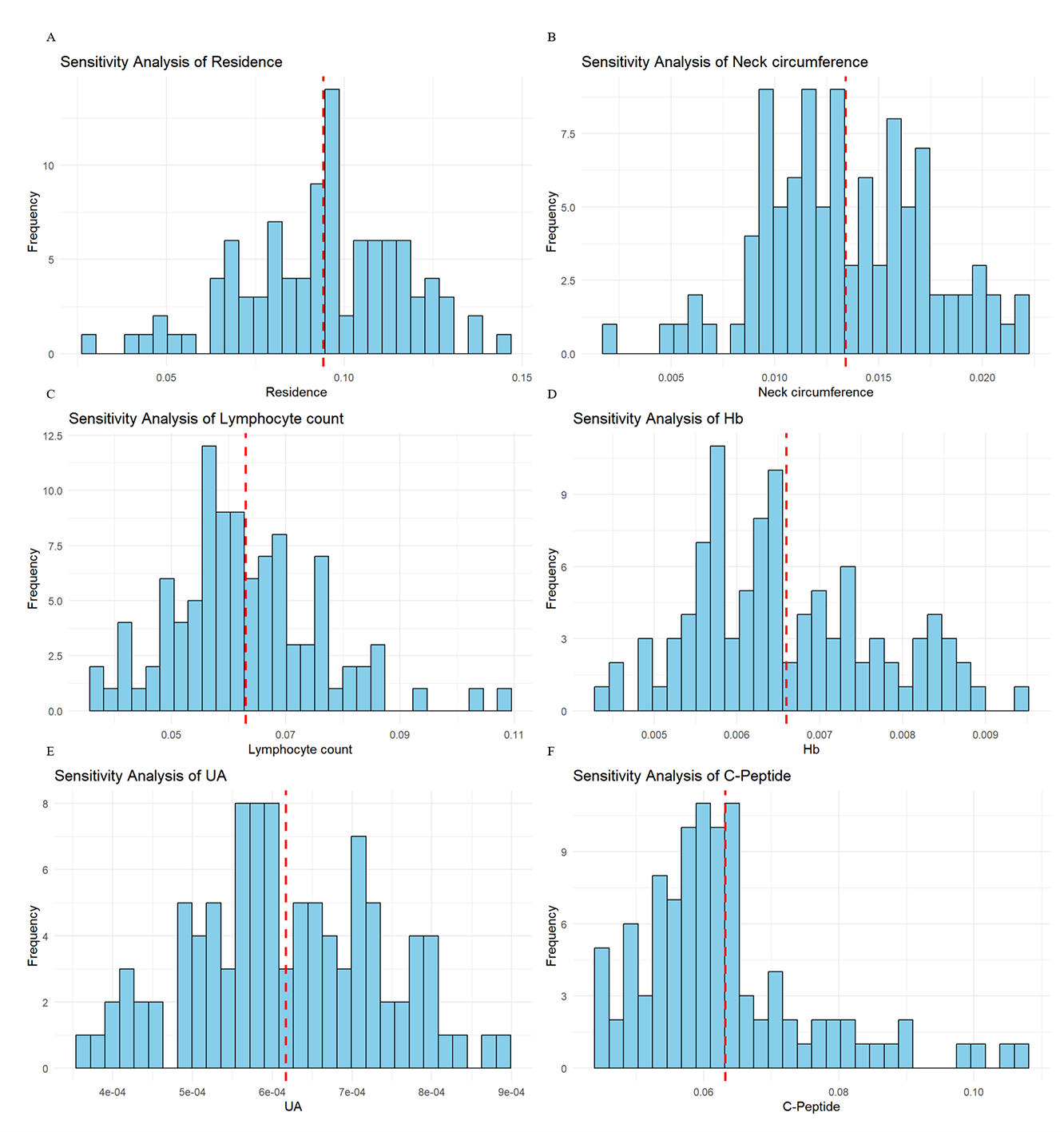

Supplement: S1 Fig — (TIF) [file pone.0334097.s001.tif]

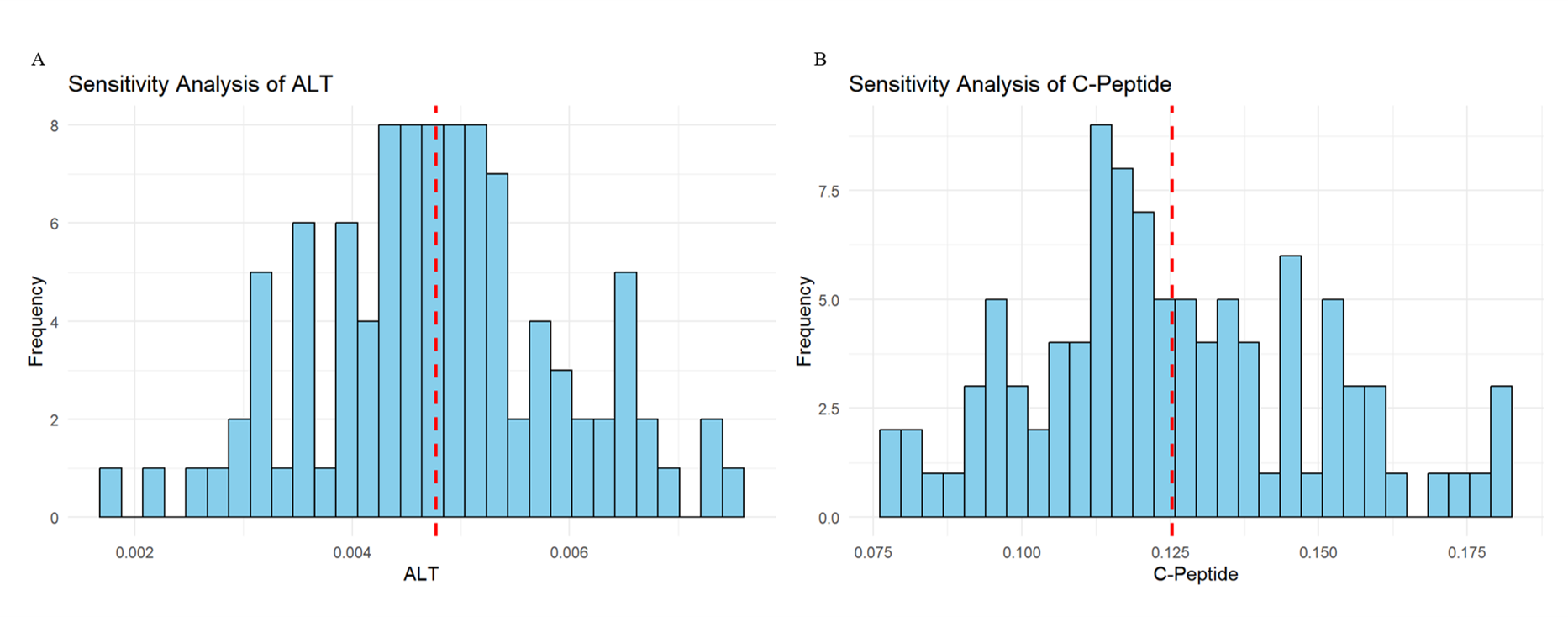

Supplement: S2 Fig — (TIF) [file pone.0334097.s002.tif]

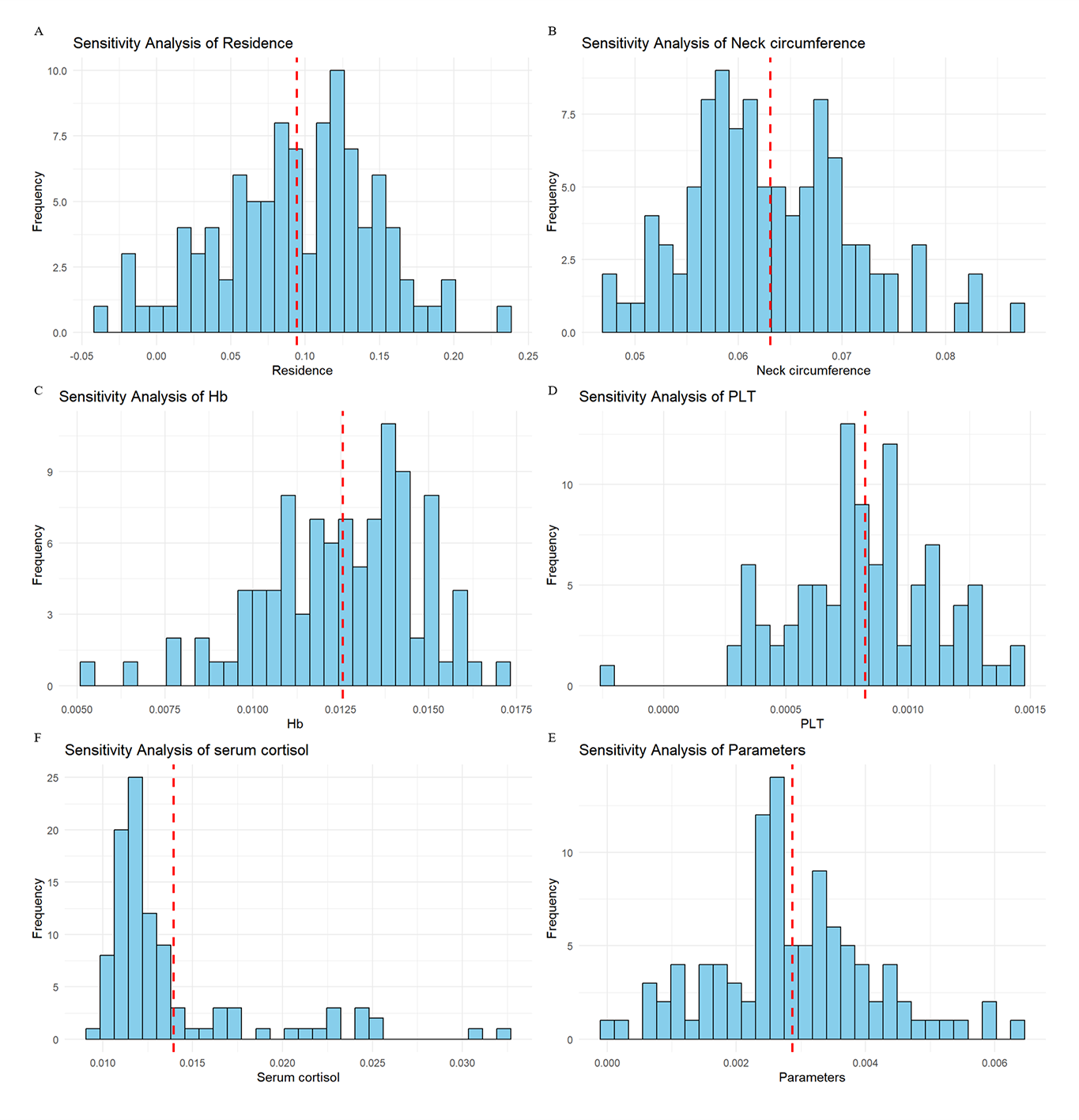

Supplement: S3 Fig — Parameters = fatty liver parameters. (TIF) [file pone.0334097.s003.tif]
